# Normal colour perception in developmental prosopagnosia

**DOI:** 10.1038/s41598-021-92840-6

**Published:** 2021-07-02

**Authors:** Chelsea Smith, Tirta Susilo

**Affiliations:** School of Psychology, Victoria University of Wellington, PO Box 600, Wellington, 6140 New Zealand

**Keywords:** Human behaviour, Perception, Neuronal development

## Abstract

Developmental prosopagnosia (DP) is a selective neurodevelopmental condition defined by lifelong impairments in face recognition. Despite much research, the extent to which DP is associated with broader visual deficits beyond face processing is unclear. Here we investigate whether DP is accompanied by deficits in colour perception. We tested a large sample of 92 DP individuals and 92 sex/age-matched controls using the well-validated Ishihara and Farnsworth–Munsell 100-Hue tests to assess red–green colour deficiencies and hue discrimination abilities. Group-level analyses show comparable performance between DP and control individuals across both tests, and single-case analyses indicate that the prevalence of colour deficits is low and comparable to that in the general population. Our study clarifies that DP is not linked to colour perception deficits and constrains theories of DP that seek to account for a larger range of visual deficits beyond face recognition.

## Introduction

Individuals with developmental prosopagnosia (DP) suffer from lifelong face-recognition deficits, despite having normal low-level vision and general intelligence^1–3^. Unlike acquired prosopagnosia, DP occurs in the absence of brain damage. Various estimates suggest that 1–2% of the population might have DP with varying severity^4–6^. DP individuals generally have little to no trouble categorising a face as a face^7^, but they are frequently unable to recognise the identity of the person it belongs to^8–11^. Some individuals also have difficulties when asked to compare the faces of unfamiliar people^11–13^, or to discriminate facial expression^14,15^, sex^16,17^, and race^18^. To compensate, DP individuals typically rely on other non-facial cues for recognition such as voice, gait, context, and general mannerisms. However, these non-facial cues have limitations and so DP individuals find social interactions challenging and disruptive to their lives^19–21^.

By diagnosis, DP is defined by selective impairments with faces. However, DP individuals can also have trouble with other kinds of visual categories^22,23^, notably everyday objects^11,24–27^, bodies^28,29^, and scenes^30,31^. A common feature of these visual categories is that their processing depends on the ventral visual pathway, which extends from the occipital lobe into inferior and lateral parts of the temporal lobe. The ventral visual pathway contains several category-selective regions for bodies, scenes, and everyday objects^32^. Research into the ventral visual pathway in DP has mainly focused on characterising the integrity of face regions^33–35^, but a recent study has looked at the integrity of both face and non-face regions and found widespread abnormality^36^. Specifically, this study found that the responses of the category-selective regions to their preferred stimuli, for both face and non-face categories, are much less selective in DP individuals than in controls. This finding supports the notion that visual impairments in DP are not restricted to face processing, and it motivates more comprehensive research into a broader range of visual functions in DP.

The human ventral visual pathway also contains regions selective for colour processing^37–39^. Flanked by face and place regions on either side, the spatial structures of these colour regions along the fusiform gyrus and collateral sulcus align with data from the macaque monkey, indicating homology between the species^39–40^. The proximity between face and colour regions in the ventral pathway suggests that face and colour processing might be more associated than previously thought. This idea accords with the human patient literature. In a meta-analysis of 92 brain-damaged cases, 70% of patients who acquired prosopagnosia also acquired colour perception deficits^41^. Further, electrical stimulation of nearby regions in the human fusiform gyrus produced real-time perceptual changes when seeing faces and coloured lights^42^.

Whether the association between face and colour deficits is present in DP is currently unknown. A recent study looked into this issue^43^ and found no evidence of impairments. However, the findings are limited in two ways. First, this study used a time-sensitive task but did not analyse response time, which raises the possibility that DP individuals might have been slower to achieve control-level performance. Second, this study tested a small sample of 9 DP individuals, which means subtle colour deficiencies and potential individual differences within the DP population might have gone undetected. Studying DP with larger samples is important since DP is a heterogenous condition with a large range of individual profiles^3,22^.

Here we report a study of colour perception with a large sample of DP individuals (N = 92) and sex/age-matched controls (N = 92). We assessed colour perception using two well-established tests for detecting colour perception deficits: the Ishihara test^44^, which is designed to pick up impairments in red–green colour discrimination, and the FM-100 test^45^, which is a sensitive test for measuring fine-grained hue discrimination abilities. We used both tests because we wish to detect any kind of colour deficits in DP. We first analysed the data to look for colour deficits at the group level, considering both accuracy and response time measures. We then assessed whether any individual DP is impaired at the single-case level.

## Methods

### Participants

We recruited 92 DP (30 males, 62 females, *M* = 39.7 years, *SD* = 9.2, range = 21–55 years) and 92 control (30 males, 62 females, *M* = 38.4 years, *SD* = 8.6; range = 24–54 years) participants. Each DP participant was sex/age-matched (within 5 years) to a control participant to help account for potential age effects in colour test performance^46^.

We sourced DP participants from Prosopagnosia Research Centre (www.faceblind.org). To satisfy DP diagnosis, participants must perform two or more standard deviations below the control mean on the 20-item prosopagnosia index^47^ (norms from Shah et al.^47^; N = 242), the Cambridge Memory Test for Faces^48^ (CFMT; norms from Duchaine and Nakayama^48^; N = 50), and a famous face test^25^ (norms from an unpublished sample; N = 189). Participants were excluded if they had a history of brain damage or other neurological problems or exhibited lower-level visual problems as detected by the Leuven Perceptual Organization Screening Test^49^ (L-POST; norms from Torfs et al.^49^; N = 200).

Control participants were sourced from Testable Minds (https://minds.testable.org/), an online service for academic researchers to recruit web-based participants. Control participants were excluded if they performed within the clinical range on the CFMT (i.e., raw score below 42). All participants provided informed consent and the study was approved by the Human Ethics Committee of the Victoria University of Wellington. The study was conducted in line with the ethical guidelines provided by the Declaration of Helsinki.

### Procedure

Our study was conducted online via Testable (www.testable.org), a web-based platform for running online cognitive and behavioural experiments. To ensure valid presentation of colour stimuli, we consulted with the Testable team and piloted the presentation across different web browsers and monitors. Colour appearance was tested using a pixel-based colour meter when presented in Chrome (for both Windows and macOS monitors) and Safari (macOS only). We found that the hue was maintained on Chrome when using a Windows PC, so we restricted participation to those with access to Chrome on a Windows PC. Before taking the tests, participants were asked to set screen brightness at maximum and calibrate stimulus size to match their screen resolution.

### Ishihara test

The Ishihara test consists of coloured pseudo-isochromatic plates that contain circles of dots and are designed to test for red–green colour blindness. The colours of the circles are strategically selected to either form a number or a line. The test version we used consists of 38 plates^50^, with 25 plates depicting a number and 13 plates depicting one or two lines, allowing for the testing of innumerate participants. We excluded the 13 plates for innumerate participants, so each participant was tested with 25 plates. Online Ishihara tests have comparable specificity and sensitivity to the traditional handbook test^51,52^.

In the test, participants were shown the 25 plates in a unique randomised order. A 300 × 300 pixel image of each plate was shown in the centre of the screen until the participant responded. Participants had to enter the number they saw on the plate or the letter “n” to indicate they did not see a number to advance to the next plate.

### Farnsworth–Munsell 100-Hue test (FM-100 test)

The FM-100 test consists of 85 isoluminant squares that vary by hue, split into four rows. Each row shows 22 squares in a random order, except for the two squares at the ends that are fixed. The square on the right end was repeated as the square on the left end of the next row. Participants were given unlimited time to arrange the squares into a continuous gradient of colour. To move a square, participants had to click on the square using a computer mouse or touchpad and drag it to a different location. Arranged correctly, the four rows can form a natural hue circle. Online FM-100 tests have shown to be effective for detecting colour perception deficits^53–56^.

In the test, each participant was presented the rows in numerical order. Only one row was shown at a time. For scoring purposes, each square had a number between 1 and 85 tracking its location in a perfect gradient. The score for each square was calculated as the sum of the absolute difference between this number and the numbers of the squares to the immediate left and right of the square, according to the participant’s arrangement. The final error score is the square’s score subtract 2. By subtracting 2, squares that are perfectly arranged have a final error score of zero. The overall performance on the test is called the Total Error Score (TES), which is the sum of the 85 square error scores. TES scores tend to be skewed, so we transformed the data for analyses by taking the square root of the TES^43,57^.

## Results

### Ishihara test

**Figure 1:**
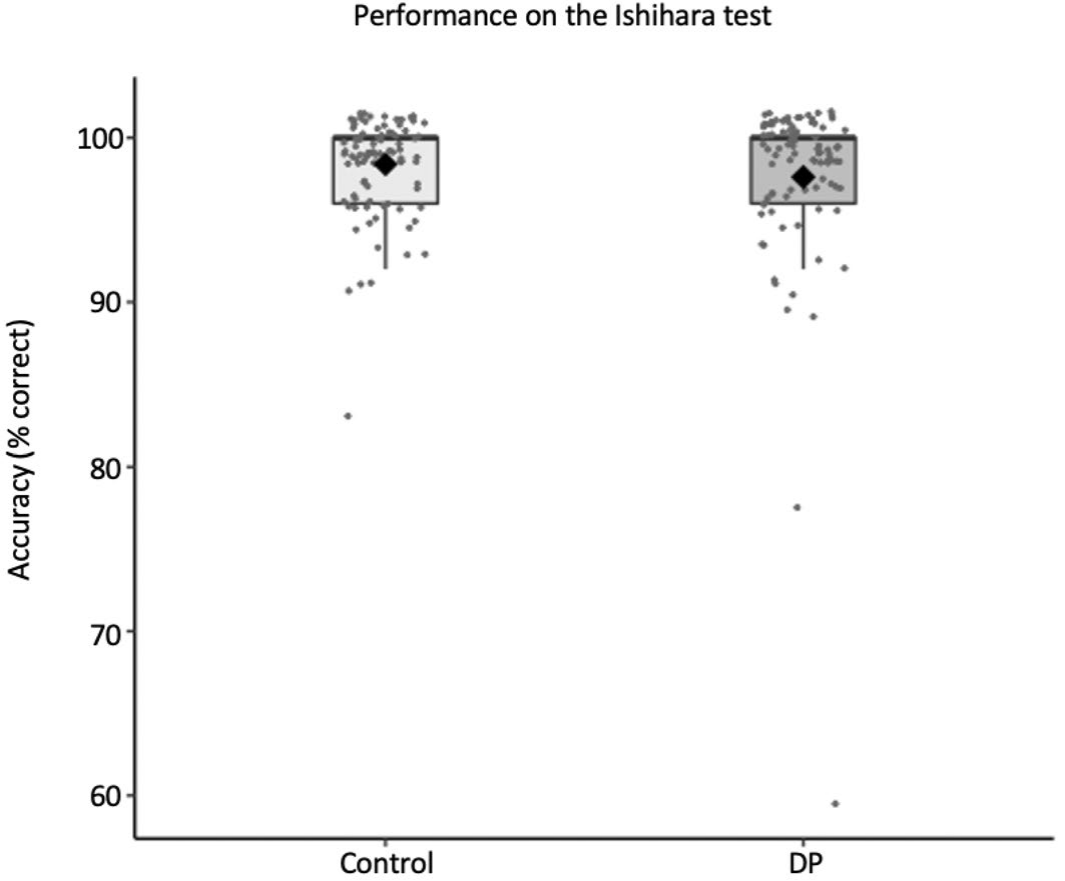
Accuracy on the Ishihara test. The upper horizontal line for each group depicts the median (100% correct for both groups). The black diamond depicts the mean.

We first compared accuracy between DP and control groups using the Mann–Whitney U non-parametric test (Fig. 1). Accuracy for DP (*M* = 97.6, *SD* = 5.4) and control (*M* = 98.4, *SD* = 2.9) groups were comparable [Mann–Whitney U = 4083, n1 = n2 = 92, *p* = .614]. Next we examined response time. DP (*M* = 1.33 min, *SD* = 0.98) and control (*M* = 1.35 min, *SD* = 0.86 min) groups completed the test with similar time [Mann–Whitney U = 3974, n1 = n2 = 92, *p* = .476]. We also performed a Bayesian t-test on the data and found moderate evidence for the null hypothesis, BF_10_ = 0.321. Together, these results show that DP as a group are not impaired on the Ishihara test.

**Figure 2:**
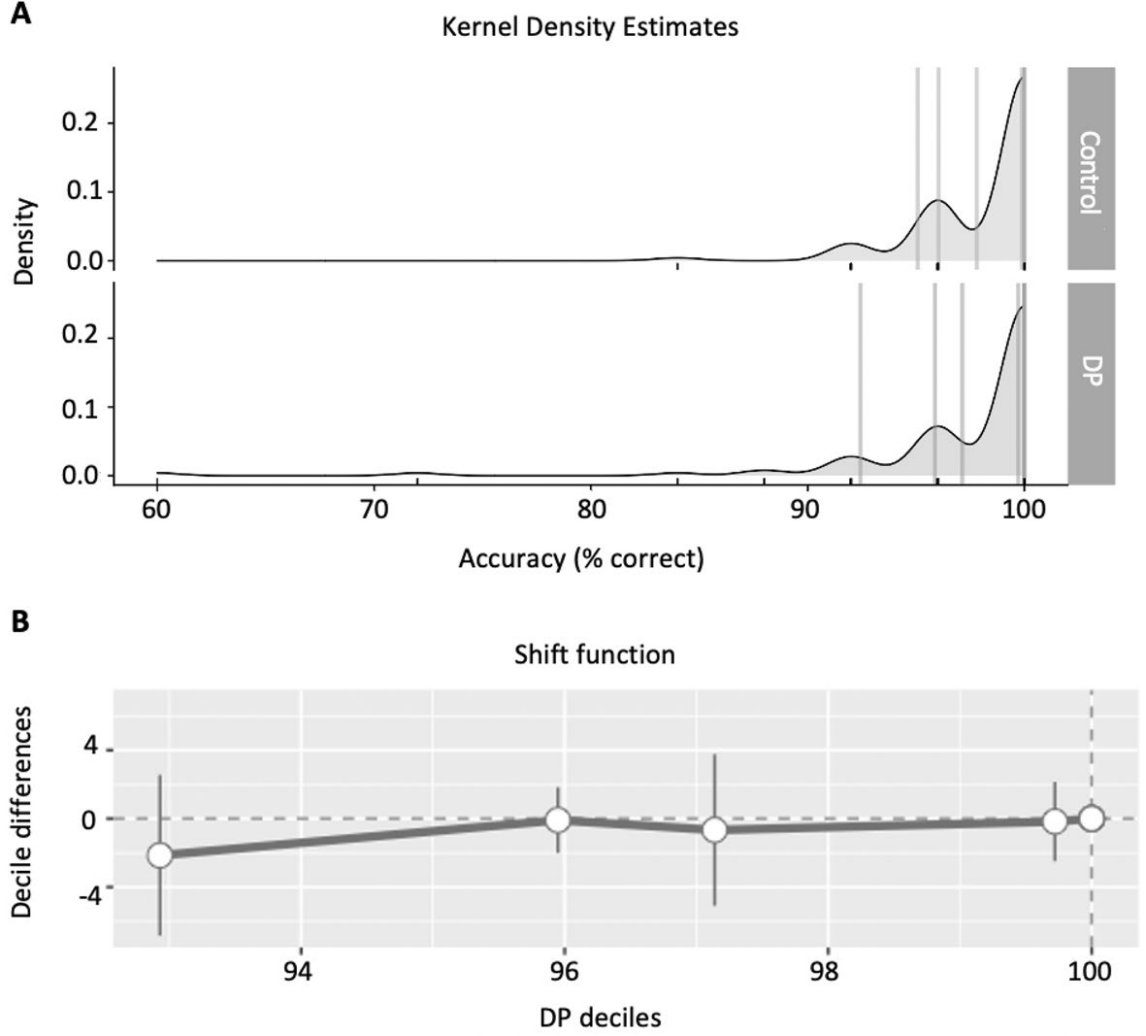
Shift function analysis on accuracy data of the Ishihara Test. Shift function plots the quantile differences of two groups as a function of the quantiles of one group. (**A**) Kernel density estimates. Group deciles are marked by vertical lines. (**B**) Shift function. The x-axis shows the deciles for DP and the y-axis shows the difference in deciles between DP and control group (DP deciles–control deciles). The vertical lines show 95% bootstrap confidence interval.

To examine the data more closely, we analysed whether the two groups performed similarly across the whole range of sample distribution. We did this using the shift function^58,59^, a graphical and inferential tool used to characterise the difference between two independent distributions (Fig. 2). Shift function plots the quantile differences of two groups as a function of the quantiles of one group. We first performed a Kolmogorov–Smirnov test to evaluate if the DP and control distributions differ overall and did not find a significant effect (test statistic = 0.043, critical value = 0.200). To create the quantiles we used the Harrell-Davis quantile estimator^60^. This process produced ten subgroups within each group (deciles), which are marked in the kernel density estimates in Fig. 2A. Figure 2B shows the deciles of the DP group on the x-axis, and the differences between DP and control deciles on the y-axis. The DP and control deciles look very similar, with all decile differences approaching zero. This analysis shows that the DP and control groups have very similar distributions of performance on the Ishihara Test.

Turning to single-case analysis, we classified performance as impaired when it falls within the 5th‰ for accuracy based on control data. Four DP individuals scored below this cut-off (scores: 88%, 88%, 72%, 60%). The two individuals scoring 88% made minor errors on three plates (e.g., entering a 6 instead of an 8) and completed the test with average time. The two individuals scoring 76% and 60% made many more errors, including substantial ones (e.g., detecting no number on a numbered plate), and one of them took an unusually long time to complete the test, falling within the 95th‰ for total test time based on control data.

Finally, we examined whether variations in face recognition skills are associated with Ishihara scores using Spearman’s correlation. We found no correlations between CFMT scores and Ishihara performance in either group (DP *r*_*s*_(90) = .14, *p* = .195; control *r*_*s*_(90) = .15, *p* = .150), as well as with pooled data from both groups (r_s_(182) = .10, *p* = .166). This result shows that face recognition and colour perception dissociate within and across both samples.

### FM-100 test

**Figure 3:**
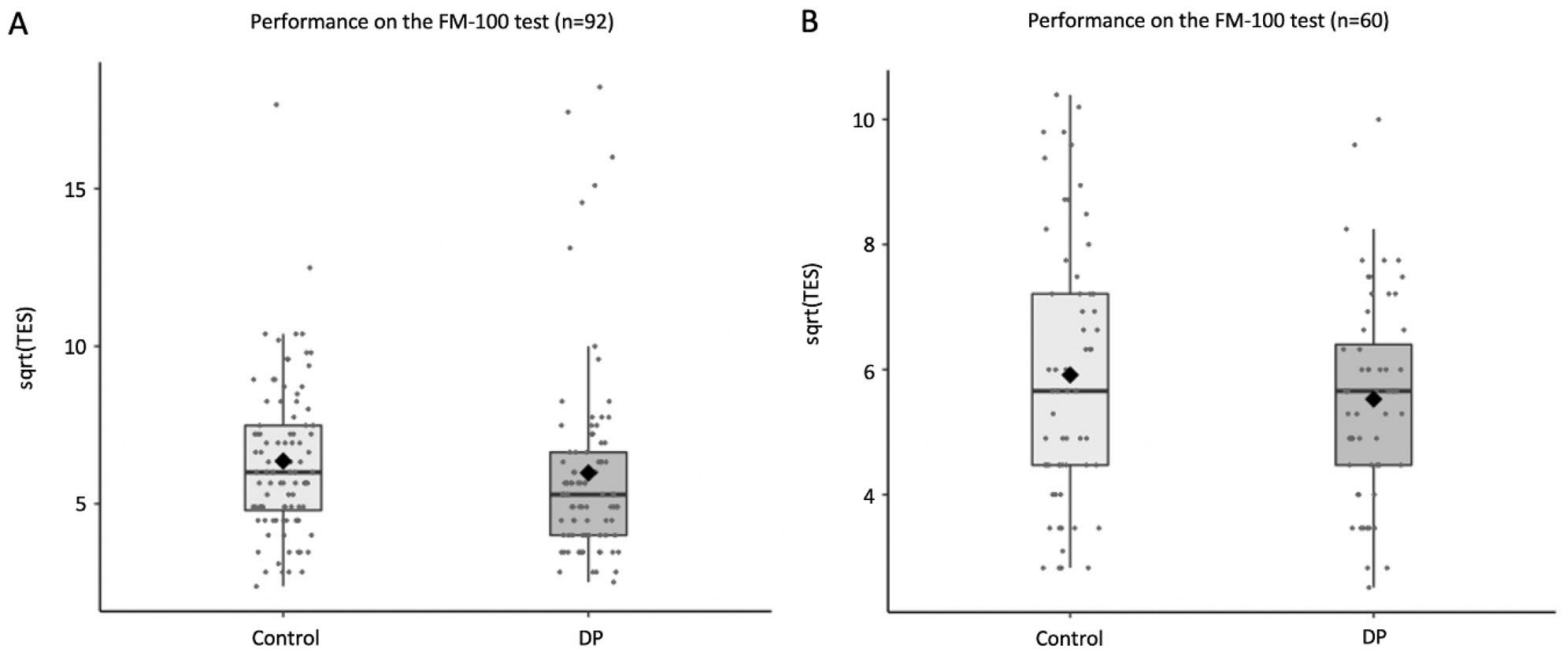
Accuracy on the FM-100 test based on the square root of the total error score. (**A**) For the whole sample. (**B**) For a subset of the sample (N = 60 in each group) with matched response time.

We first performed a square root transformation on the TES data since the data was skewed for both groups (DP = 3.40, control = 3.54) as advised by Kinnear^57^ and Moroz et al.^43^. The transformation successfully reduced skewness for both groups (DP = 2.32, control = 1.32). We also applied a separate logarithm transformation on the data, and it yielded similar results to the square root transformation. We used the Mann–Whitney U non-parametric test to compare accuracy between DP and control groups as indexed by sqrt(TES) (Fig. 3A). The DP group showed lower sqrt(TES) (*M* = 5.98, *SD* = 3.04) than controls (*M* = 6.35, *SD* = 2.44), [Mann–Whitney U = 3493, n1 = n2 = 92, *p* = .040], indicating slightly more accurate sorting. However, the DP group also took more time to complete the test (*M* = 9.25 min, *SD* = 5.37 min) than controls (*M* = 8.58 min, *SD* = 4.06 min), [Mann–Whitney U = 3493, n1 = n2 = 92, *p* = .04], suggesting a speed-accuracy trade-off. We additionally performed a Bayesian t-test on the data and found moderate evidence for the null hypothesis, BF_10_ = 0.238.

To address this issue, we ran two analyses. First, we performed an ANCOVA to compare sqrt(TES) between the groups with response time as a covariate. This analysis revealed no group difference [F(1,181) = 0.526, *p* = .469], and that response time explained a substantial variance between the two groups [F(1,181) = 10.07, *p* = .002]. A Bayesian ANCOVA revealed similar results (BF_incl_ = 0.213 for group effect; BF_incl_ = 18.7 for response time). Next, we analysed a subset of the DP and control samples that were matched on response time (Fig. 3B). We matched as many participants as possible across the groups with a response time difference of + /− 15 s, such that each DP participant was matched to a control participant with a nearly identical response time. This process resulted in 60 DP (M = 8.66 min, SD 3.80 min) and 60 control (M = 8.67 min, SD 3.81 min) participants. With this subset, we again found no sqrt(TES) difference between the groups [t(118) = 1.13, *p* = .262]. Overall, these analyses show that DP as a group are not impaired on the FM-100 test.

**Figure 4:**
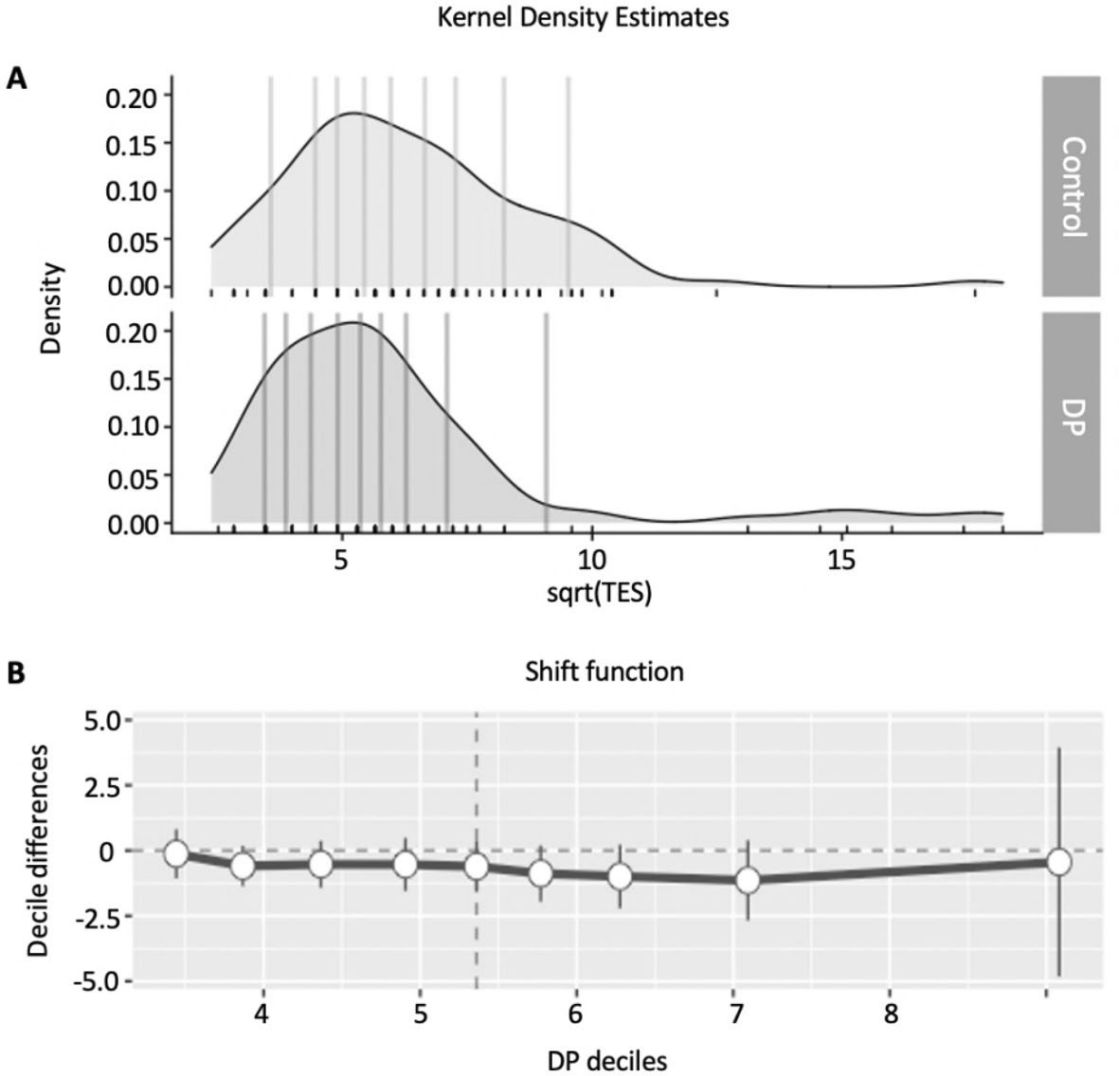
(**A**) Kernel density estimates. Group deciles are marked by the vertical lines. (**B**) Shift function. The x-axis shows the deciles for DP individuals and the y-axis shows the difference in deciles between DP individuals and controls (DP individual deciles–control deciles). The vertical line for each decile difference specifics its 95% bootstrap confidence interval.

To delve deeper into the results, we examined sqrt(TES) across the whole range of sample distribution using the shift function (Fig. 4). A Kolmogorov–Smirnov test revealed that the DP and control distributions were overall similar (test statistic = 0.163, critical value = 0.200). The Harrell-Davis quantile estimator^60^ split each group into deciles, and the figure shows that all decile differences approached zero. This means DP and control groups performed similarly on the FM-100 test across the whole sample distributions.

We also explored the data at the single-case level. We classified performance as impaired when it falls within the 95th‰ for sqrt(TES) and 95th‰ for response time based on control data. Six DP individuals fell outside the range for sqrt(TES), but five had response time similar or faster (between 3.8 and 5.9 min) than the control mean (8.6 min), making interpretations difficult. Only one individual came out abnormal on both sqrt(TES) and response time.

Finally, we tested whether variations in face recognition skills predict scores on the FM-100 test using Spearman’s correlation. We found no correlations between CFMT scores and FM-100 scores in either group (DP *r*_*s*_(90) = − .15, *p* = .164; control *r*_*s*_(90) = − .16, *p* = .128) and across both groups (r_s_(182) = .06, *p* = .461), again suggesting that face recognition and colour perception are dissociated abilities.

### Validation of online tests

Our analyses of both tests found no deficits of colour perception in DP. This raises the possibility that our online tests might not have been sensitive enough to detect colour deficits. To address this issue and further validate our online approach, we asked a group of five colour-blind participants (4 males, 1 female, *M* = 29.8 years, *SD* = 15.7) to take both tests. All participants are aware of their colour deficiencies and have been diagnosed with colour blindness. All five individuals performed in the clinical range. On the Ishihara test, the accuracies (range: 52–84%) fell below the 5th‰ cut-off (92%). Similarly on the FM-100 test, the scores (range: 8.3–20.0) fell above or near the 95% percentile cut-off (10.2). This validation analysis shows that our online colour tests were able to detect colour deficiencies, and that the null results of our DP study cannot be explained by methodological issues.

## Discussion

In this study we addressed whether colour perception is impaired in DP. We tested 92 DP individuals and 92 controls on the Ishihara and FM-100 tests to evaluate red–green colour deficiencies and hue discrimination abilities. At the group level, DP and control participants performed similarly on accuracy. This finding holds when response time was considered, and when we examined the score distribution across the whole sample with shift function analyses. At the single-case level, there is some evidence of colour perception impairment in a small number of DP individuals. The Ishihara test suggested 4 DP individuals showed weak red–green colour discrimination, although two of these individuals showed minor errors in their responses. The FM-100 test indicated a different group of 6 DP individuals had abnormal hue discrimination scores. However, when considering the potential speed-accuracy trade-off, only one of these DP individuals also fell out of the normal response time range. In addition, performance on the Cambridge Face Memory Test (CFMT) did not predict performance on either colour test in either group, further suggesting that colour discrimination and face perception are dissociated. Overall, our study demonstrates that face recognition deficits in DP are not associated with broader visual deficits in colour perception.

Our group results align with and extend that of Moroz et al.^43^, which reports normal FM-100 performance in 9 DP individuals. Our much larger sample size enhances the robustness of the conclusion that hue discrimination abilities are normal in DP, and our Ishihara results further clarify that DP is not linked to red–green colour deficiencies. A key contribution of our study is the analysis of response times, which is particularly important for a time-sensitive test such as FM-100. Another contribution is the shift function analyses, which provide a closer look at the distribution of performance across the whole sample. These analyses together bolster the main conclusion that colour perception is not impaired in DP.

A small minority of DP individuals showed abnormal performance at the single-case level. Four individuals came up impaired on the Ishihara Test—two with minor errors (e.g., entering a 6 instead of an 8) and two with substantial errors (e.g., not identifying any number on a numbered plate). For the FM-100 test, six individuals showed impaired accuracy, but three of them completed the test in about half the average control time, suggesting potential speed-accuracy trade-offs. Only one individual had an impaired FM-100 accuracy as well as a completion time that is abnormally long compared to the control average. Interestingly, this individual also made one minor error in the Ishihara Test (entered 8 instead of 9), although this is not uncommon (19/92 DP individuals and 21/92 controls made one error). In sum, considering the two individuals with major Ishihara errors and the one individual with clear FM-100 deficit, the prevalence of colour perception problems in DP seems very low (around 3%) and not elevated compared to the prevalence in the general population (around 4–8% for males and around 0.4% for females)^61,62^.

Our study provides strong evidence that helps clarify the extent of broader visual deficits in DP. Research into this issue has mainly focused on testing visual recognition of non-face objects^24,25,28,63–66^, but a growing number of studies has started to assess a broader range of visual functions including biological motion^10,67^, navigation^30,31,68^, word processing^69^, and colour vision^43^. These studies tend to find mixed results^22,23^, likely due their small sample sizes and the potentially heterogenous nature of DP. Small-sample studies risk generalising particular findings that may apply to a potential subgroup of DP rather than DP as a whole. Against this backdrop, our large-sample study suggests that our findings on colour perception are robust to the DP population. Our large sample also permitted multi-level analyses across the whole group, subgroups (i.e., group deciles) and individual cases. Across all levels, our results consistently show that colour impairment is not more common in DP compared to the typical population. This finding indicates that, in the case of colour perception, DP does not appear to be heterogenous. Our finding aligns with the theoretical view that DP is a domain-specific disorder of face processing^29,63^, and that non-face deficits in DP likely result from independent impairments that may co-vary with DP. Our finding also puts a constraint on more generalist theories that seek to explain DP in terms of broader visual impairments beyond face processing^22^, by requiring those domain-general models to account for normal colour perception.

Our findings may provide insight into the typical organisation of face and colour processing systems. To the extent that architectures of normal cognition can be inferred from studies of selective developmental conditions^70,71^, our results suggest that face and colour processing systems can develop and function independently. Our study also implies that face and colour deficits in brain-damaged cases often co-occur because injury to the ventral visual pathway tend to be diffused and therefore impact multiple visual functions, not because face and colour processing share the same neural circuits. This insight accords with a recent intracranial stimulation study in a neurological patient that found a double dissociation between face and colour perception in nearby sites of the fusiform gyrus^72^.

A potential concern about our study is the use of online testing. Online testing has become mainstream in many areas across psychology and cognitive science^73–75^, including in colour perception^39,76,77^. However, there remains the inevitable lack of control over various stimulus parameters (e.g., stimulus size, brightness of screen, colour appearance, monitor type). To mitigate these concerns we took several steps, including restricting participation to PC monitors and Chrome browsers that are optimal for colour presentation based on our piloting, instructing participants to calibrate stimulus size to their screen resolution, and, most critically, collecting control data in the same way as we did DP data. While we cannot rule out potential differences in testing parameters across individual participants (such as the use of different monitors by different individuals), we have no reason to suspect that these differences would occur systematically between the two groups to the extent that they may account for our findings. Generic issues related to online testing would likely be washed out when comparing data across large-sample groups that were tested in identical manner, which is what we focused on in our analysis. Finally, our validation data with the colour-blind participants showed that our online tests are able to detect colour perception deficits in individuals who have them.

Another potential concern about the study is the lateralisation of neural abnormalities in DP. Most imaging studies suggest that DP individuals tend to have bilateral abnormalities^33,36^, but some may have unilateral abnormalities^78^. It is possible that in the case of unilateral abnormalities, colour perception deficits may only be detected by careful testing in the corresponding hemifield. Future studies testing colour perception in each hemifield would address this issue and help clarify whether our study underestimates colour perception deficits in DP because our testing was performed in central vision.

Finally, our findings and conclusion are limited to basic colour perception. Future studies should examine other aspects of colour processing beyond colour perception. One aspect is colour knowledge. In brain-damaged cases, impairments in retrieving an object’s prototypical colour (i.e., colour agnosia) tend to result from lesions more anterior in the ventral visual pathway than impairments of colour perception (i.e., achromatopsia), which tend to result from more posterior lesions^79^. Double dissociations between colour agnosia and achromatopsia have been reported^80,81^, raising the possibility that while colour perception is normal in DP, colour knowledge may not be. Another aspect worth investigating is processing of colour that is more specific to faces, such as skin colour and eye colour^82–84^. For example, one study found impaired performance on eye colour perception by two DP individuals who did not seem to have general colour deficits (Barton et al.^85^). Characterising other aspects of colour processing beyond basic colour perception will provide a more complete picture of colour vision in DP.
